# Expression and prognostic value of APOBEC2 in gastric adenocarcinoma and its association with tumor-infiltrating immune cells

**DOI:** 10.1186/s12885-023-11769-3

**Published:** 2024-01-02

**Authors:** Lipan Wei, Xiuqian Wu, Lan Wang, Ling Chen, Xuejun Wu, Tiantian Song, Yuanyuan Wang, Wenjun Chang, Aizhen Guo, Yongdong Niu, Haihua Huang

**Affiliations:** 1 Department of Pathology, Second affiliated Hospital of Medical College of Shantou University, Shantou, China; 2 Department of Interventional Oncology, Cancer Hospital of Shantou University Medical College, Shantou, China; 3 Department of Pharmacology, Shantou University Medical College, Shantou, China; 4 Department of Pathology, Shantou Central Hospital, Shantou, China; 5 Department of Environmental Hygiene, Second Military Medical University, Shanghai, China; 6 Department of General Practice, Yangpu Hospital, School of Medicine, Tongji University, Shanghai, China

**Keywords:** APOBEC2, CD66b, Gastric cancer, Adjuvant chemotherapy, Prognosis

## Abstract

**Background:**

Apolipoprotein B mRNA editing enzyme catalytic polypeptide-like 2 (APOBEC2) is associated with nucleotide alterations in the transcripts of tumor-related genes which are contributed to carcinogenesis. Expression and prognosis value of APOBEC2 in stomach adenocarcinoma (STAD) remains unclear.

**Methods:**

The APOBEC2 gene alteration frequency of STAD and APOBEC2 gene expression in STAD and normal tissues were investigated in cBioportal and GEPIA, respectively. We detected expression of APOBEC2, infiltration of CD66b^+^ tumor-associated neutrophils and CD163^+^ tumor-associated macrophages in tissue microarrays by immunohistochemistry. APOBEC2 gene expression was explored by western blot and qRT-PCR. Relationships between APOBEC2 and CD66b, CD163, and other clinicopathological characteristics were investigated. Associations among APOBEC2 expression status and patient survival outcome were further analyzed.

**Results:**

APOBEC2 gene alteration frequency was 5%, and APOBEC2 gene was downexpressed in STAD compared to normal tissues (*P* < 0.05). APOBEC2 expression status were associated with the infiltration of CD66b^+^ TANs, differentiation grade, TNM stage, histological type and gender (all *P* < 0.05) in STAD. Little or no APOBEC2 expression was detected in STAD and adjacent normal tissues by western blot. We failed to show that APOBEC2 was an independent risk factor for OS (Hazard Ratio 0.816, 95%CI 0.574–1.161, *P* = 0.259) or DFS (Hazard Ratio 0.821, 95%CI 0.578–1.166, *P* = 0.270) in STAD by multivariate Cox regression analysis, but APOBEC2 negative subgroup has a worse OS and DFS among patients with adjuvant chemotherapy.

**Conclusions:**

APOBEC2 correlates with CD66b, differentiation grade, TNM stages, histological classification, and gender in STAD. APOBEC2 is not an independent prognostic factor for STAD, our results suggest that patients with positive APOBEC2 can benefit from postoperative chemotherapy, and combination of APOBEC2 and CD66b is helpful to further stratify patients into different groups with distinct prognoses.

**Supplementary Information:**

The online version contains supplementary material available at 10.1186/s12885-023-11769-3.

## Introduction

Gastric cancer is the fifth most common cancer and the fourth cancer death cause globally, with over 1 million new cases diagnosed and about 777,000 deaths in 2020 [1]. In China, the number of newly diagnosed with gastric cancer (GC) patients is nearly 500,000, and the morbidity and mortality of gastric cancer occupies the third position in malignant tumors [2–3].

Over the past decade, some advancements have been made in the immunotherapy of gastric cancer, but the low immunogenicity of tumors, tumor-induced immunosuppression, drug resistance, and toxic effects all limited the application of immunotherapy in clinical practice [4–5]. Therefore, it is important to identify more biomarkers and therapeutic targets that can be used as clinical markers for GC clinical diagnosis, prognosis, chemotherapy, and tumor immunotherapy.

APOBEC2 is the second member of APOBEC family, and the protein family is known to mediate editing of the C (cytidine)→U (uridine) by cytidine deaminating activity and contributes to the mutagenesis of genomic DNA [6]. Recent observations have also linked the expression of APOBEC2 to tumors and indicated it in the regulation of tumor-related genes, including Eif4g2 and PTEN [7–9]. Kostic et al. reported that the APOBEC2 gene is a target gene of TP53, while TP53 frequently mutates in GC [10–11]. Based on genomic data of The Cancer Genome Atlas (TCGA), Shi et al. found that APOBEC2 transcriptional level was significantly correlated with the tumor mutational burden (TMB) in stomach adenocarcinoma (STAD) [12]. In addition, APOBECs have been known to induce immunogenicity by mediating hypermutation and enhance immunological-related markers such as immune cell infiltration [13–15]. However, the significance of APOBEC2 protein expression in GC, especially in its association with tumor-infiltrating immune cells is unknown. CD66b as a phenotype of high-density or low-density neutrophils, which has been found to expressed in human tumor-associated neutrophils [16].

In this study, we investigated the APOBEC2 gene mutation in STAD in the publicly available clinicogenomic data on cBioportal. We also explored the transcription level of APOBEC2 in STAD and normal tissues from the GEPIA database. Western blot and qRT-PCR were used to detect APOBEC2 expression in STAD and normal tissues. Finally, we used immunohistochemistry to assess the expression of APOBEC2, CD66b and CD163 in tissue microarrays (TMAs) from 496 patients with STAD. The relationship of APOBEC2 with CD66b, CD163, and other clinicopathological characteristics was investigated. The associations between APOBEC2 expression and patient survival outcome were further analyzed.

## Materials and methods

### Identifying APOBEC2 genetic alteration in cBioportal

An open-access cancer genomics platform, the cBio Cancer Genomics Portal (http://www.cbioportal.org/), gathers multidimensional cancer genomics data sets from the Gene Expression Omnibus (GEO), the International Cancer Genome Consortium (iCGC), TCGA and other databases [17]. The cBio Cancer Genomics Portal empowers researchers to explore genomic data such as DNA copy number alterations (CNAs), DNA methylation and somatic mutations in an intuitive and rapid way. There are seven gastric adenocarcinoma-related studies including 1,512 samples at the time of this writing. Since some patients contributed multiple samples to these studies, we excluded 735 overlapping cases and explored DNA CNAs of APOBEC2 gene in the remaining 777 cases.

### Exploring APOBEC2 gene expression in GEPIA

GEPIA (http://gepia.cancerpku.cn/) is a publicly accessible website that offers visualization of gene differential expression, patient survival analysis, profiling plotting, and relevant genomic information based on Genotype Tissue Expression (GTEx) and TCGA data [18]. We analyzed the expression of APOBEC2 gene in gastric adenocarcinoma (n = 408) and adjacent tissues (n = 211) using the function of box plots in the GEPIA database.

### Detecting of APOBEC2 protein in cancer and paracancerous tissue microarrays

The medical records of patients with gastric cancer who received gastrectomy at the First Affiliated Hospital of the Second Military Medical University from December 2006 to July 2011 were evaluated [19]. Patients who received preoperative cancer treatment and were diagnosed with autoimmune diseases or other malignant tumors were excluded. Finally, 496 Patients with gastric adenocarcinoma were enrolled in this study, and their formalin-fixed paraffin-embedded (FFPE) tissue blocks were collected and made into TMAs. The TMAs contained 496 cancer tissues and 48 non-malignant tissues (constructed by Outdo Biotech, shanghai, China). The hematoxylin-eosin (H&E) slides of 496 patients were also collected. The clinical and pathological information of 496 patients, including age, gender, serum CA199 (carbohydrate antigen 199) level, serum CEA (carcinoembryonic antigen) level, tumor size, differentiation, WHO histological classification, Lauren’s histological type, and TNM stage, was gathered from the electronic medical record and histologic sections (Table 1). The tumors were classified as follows according to the 7th edition of the American Joint Committee on Cancer Staging Manual: Stage I (n = 160), Stage II (n = 134), Stage III (n = 195), and Stage IV (n = 7). Of these, 339 patients received postoperative chemotherapy. In this research, the median follow-up duration was 60 (1-101) months. Overall survival (OS) was defined as the date of surgery to the last follow-up date or death, and disease-free survival (DFS) was defined as the date of surgery to the time of recurrence or metastasis.

### Immunohistochemical staining and scoring

The antibodies we used as the following: APOBEC2 (dilution 1:100, rabbit anti-human APOBEC2 monoclonal antibody, ab170859, Abcam), CD66b (dilution 1:400, no.555,723, BD Biosciences, NJ), CD163 (ready-to-use, MAB-0206, Maxim, Fuzhou, Republic of China), secondary antibodies (MaxvisionTM2 HRP-polymer anti-mouse/rabbit IHC kit, Fuzhou, China).

The immunohistochemical EnVison two-step method was applied to detect the expression of APOBEC2. Details of Immunohistochemical APOBEC2 staining were presented in Fig. S1 (see Appendix). The positive expression of APOBEC2 was localized to cytoplasm. The staining intensity was scored as follows: 0 point for negative staining, 1 point for light yellow, 2 points for moderate yellow, 3 points for brown. If the percentage of cells is more than 75%, it scores 3 points; 51–75%, 2 points; 10–50%, 1 point; and below 10%, nil. An immunohistochemical score was calculated as the score for staining intensity plus the score for percentage of cells. The final score of IHC greater than 1 was regarded as positive, and a score equal to or less than 1 was regarded as negative. Immunohistochemistry protocols for CD66b and CD163, and quantitative evaluation of immunostaining, were presented in a previous study [19]. For survival and correlation analysis, patients were divided into CD66b^low^, CD66b^high^, CD163^low^ and CD163^high^ groups, using the median value as a cut-off.

### Western bolt and real-time quantitative reverse transcription PCR (qRT-PCR)

Homogenates of skeletal muscle, STAD and adjacent normal tissues were diluted in 5×loading buffer and boiled for 5 min. 20 µg/lane protein samples were electro-transferred onto polyvinylidene fluoride after being resolved by 10% sodium dodecyl sulfate-polyacrylamide gel electrophoresis. The primary antibodies used as the following: APOBEC2 (dilution 1:400, rabbit anti-human APOBEC2 monoclonal antibody, ab170859, Abcam) and α-Tubulin (dilution 1:10000, mouse monoclonal antibody, ab7291, Abcam).

Total RNA was extracted from cell (gastric cancer cell line HGC27 and AG5), STAD and normal gastric tissues using Trizol (Takara, Japan) and the concentration was determined by Nanodrop. RNA was reverse transcribed into cDNA using PrimeScript RT kit with gDNA Eraser (Takara, Japan) at room temperature for 5 minutes, 37˚C for 30 minutes and 85˚C for 5 seconds. Using Applied Biosystems 7500 Real Time PCR system, real-time PCR was conducted as follows: 95˚C for 2 minutes; 40 cycles of 95˚C for 10 seconds and 60˚C for 30 seconds; 95˚C for 15 seconds; 60 ˚C for 1minutes; 95˚C for 15 seconds at the last step. The primer sequences we used were as follows: APOBEC2, forward 5’-CCTCTCTCCTCTCCCTCAGT-3’, reverse 5’-TTTCAGCTTCTCAGGGTCGT-3’; CYCLOPHILIN, forward 5’-TGGTGTTTGGCAAAGTGAAA-3’, reverse 5’-TCGAGTTGTCCACAGTCAGC-3’ (Sangon Biotech, China).

### Statistical analysis

To compare the APOBEC2 expression between cancer samples and adjacent gastric samples, Kruskal–Wallis test or paired t-test was performed with GraphPad Prism 8.2. By using SPSS21.0 software (IBM SPSS Statistics for window, Version 21.0), the Mann-Whitney U test and Pearson Chi-square test were used to analyze the association between APOBEC2 expression and clinicopathological variables of patients. The Kaplan-Meier curves and Log-rank test was performed to analyzed differences in survival outcomes using with GraphPad Prism 8.2. To identify prognostic factors of OS and DFS, the univariate and multivariate Cox proportional hazards regression models were applied using SPSS21.0 software. *P* value < 0.05 was regarded as statistical significance.

## Results

### APOBEC2 gene mutation, amplification and deletion was observed in STAD

**Fig. 1:**
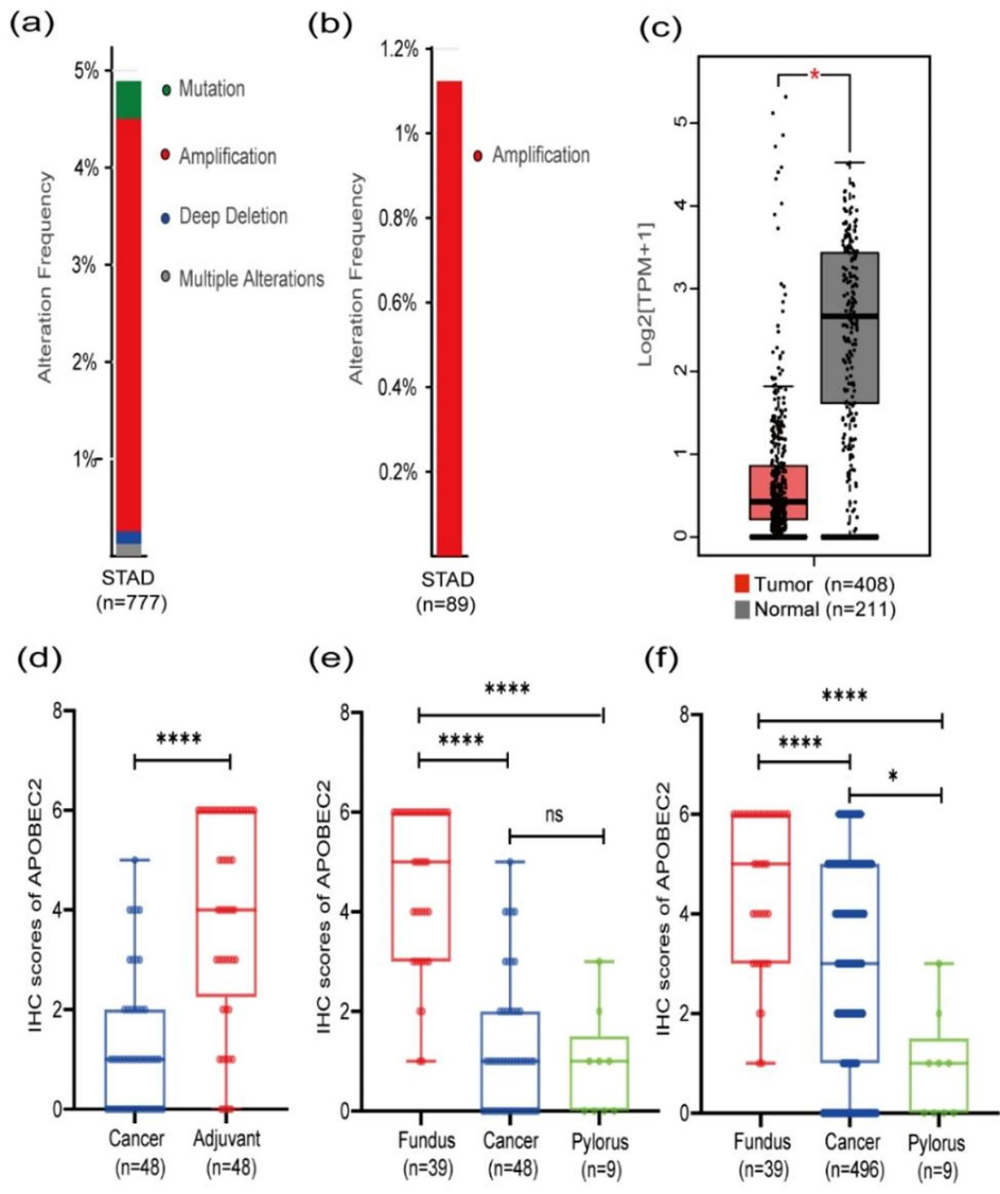
APOBEC2 genetic alteration, transcripts, and protein expression pattern in STAD and adjacent tissues. (**a**) APOBEC2 genetic alteration (mutation, amplification, deep deletion and multiple alteration) in 777 STAD. Data were obtained from cBioPortal and shown by composition ratio. (**b**) APOBEC2 genetic amplification in 89 Asians STAD. Data were obtained from cBioPortal and shown by composition ratio. (**c**) Box plots of comparing detail APOBEC2 transcripts between normal and STAD. Data were obtained from GEPIA and the method for differential analysis is one-way ANOVA. (**d**) Comparison of IHC score of 48 paired malignant and non-malignant specimens. (**e**) Comparison of IHC score of 39 fundic gland mucosa tissues, 48 cancer tissues and 9 pyloric gland mucosa tissues. (**f**) Comparison of IHC score of 39 fundic gland mucosa tissues, 496 cancer tissues and 9 pyloric gland mucosa tissues. Paired t-test was used in Fig. 1d. The Kruskal–Wallis test was used in Fig. 1e and f. *P < 0.05, ****P < 0.0001, ns (no significant)

We investigated the frequency of APOBEC2 gene alteration using the TCGA dataset, OncoSG dataset, UHK dataset, U Tokyo dataset, and Pfizer & UHK dataset from cBioPortal. A total of five stomach adenocarcinoma studies including 777 patients (of which 89 were Asian) was shown in cBioPortal, while those with repetitive studies were excluded. The results obtained from cBioPortal suggested that the proportion of APOBEC2 gene alteration was about 5% (38/777, Fig. 1a). Among these only 1 of 89 Asian existed APOBEC2 gene alteration (Fig. 1b). In TCGA dataset (STAD; TCGA Firehose Legacy, cBioPortal), the proportions of APOBEC2 gene mutation, copy number amplification, deep deletion and multiple alteration were 0.42% (2 in 478 cases), 3.97% (19 in 478 cases), 0.21% (1 in 478 cases), and 0.21% (1 in 478 cases), respectively. In the OncoSG dataset (STAD; OncoSG dataset, cBioPortal), the proportions of APOBEC2 gene mutation, and copy number amplification were 0.68% (1 in 147 cases) and 9.52% (14 in 147 cases), respectively.

### Differential APOBEC2 gene expression in STAD and adjacent tissues

We utilized the TCGA and GTEx datasets from GEPIA to explore the level of APOBEC2 mRNA in stomach adenocarcinoma tissues and adjacent non-malignant tissues. It showed that APOBEC2 transcripts were found to be significantly down regulated in stomach adenocarcinoma tissues compared to normal gastric tissues (*P* < 0.05; Fig. 1c).

### Expression pattern of APOBEC2 protein in STAD and adjacent tissues

**Fig. 2:**
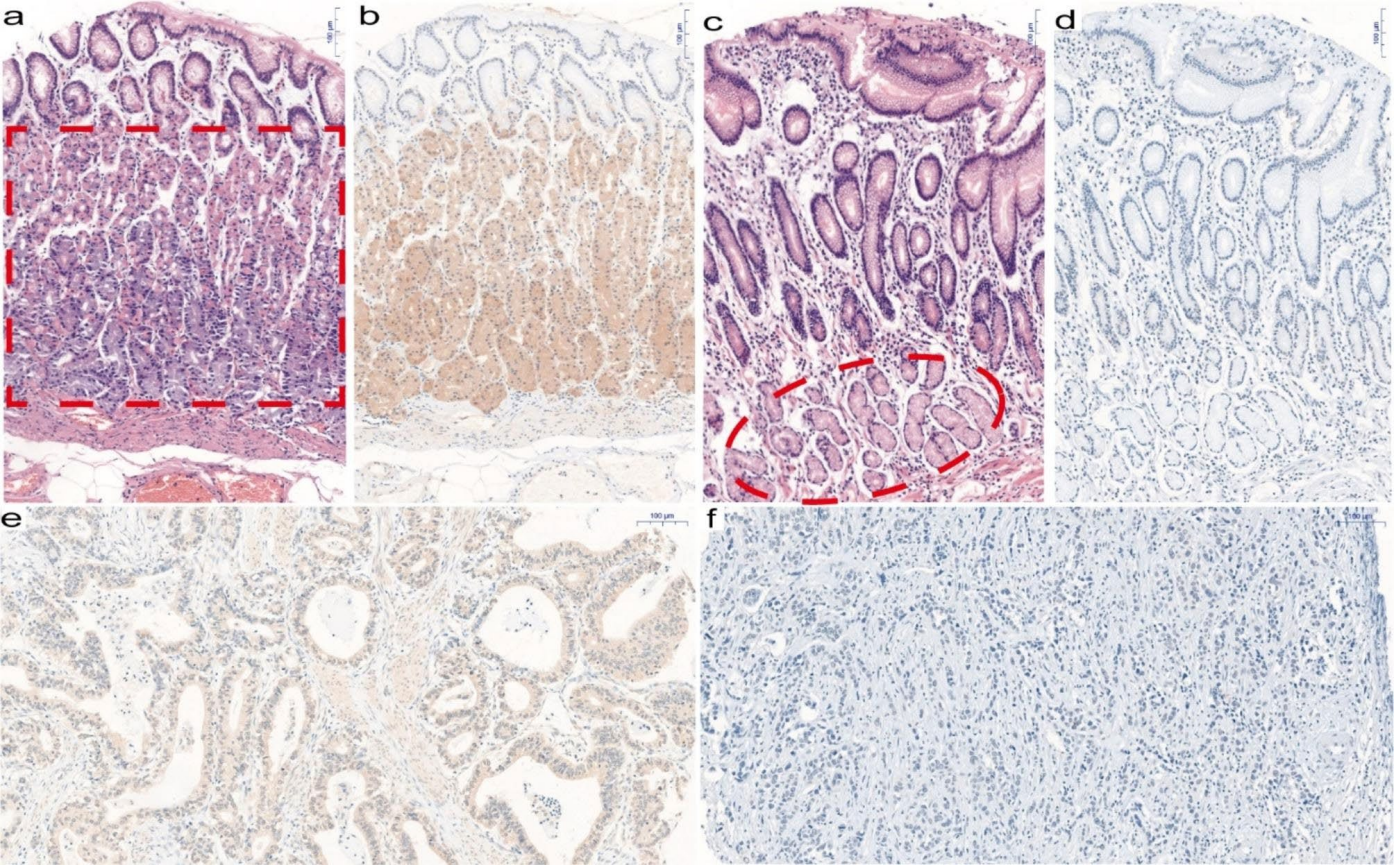
Representative immunohistochemistry images of APOBEC2 staining in STAD and adjacent tissues. (**a**) Parietal cells predominating in the upper part of the glands and chief cells predominating in the basal regions of the glands (H&E). (**b**) Parietal cells and chief cells showing APOBEC2 expression. (**c**) Pyloric glands consisting of mucous glands (H&E). (**d**) Negative expression of APOBEC2 in the pyloric gland. (**e**) Positive expression of APOBEC2 in STAD. (**f**) Negative expression of APOBEC2 in STAD. Scale bar: 100 μm

The immunohistochemical staining findings, obtained for 496 malignant and 48 non-malignant tissues, showed that APOBEC2 was mainly located in the epithelial while rarely in stromal cells. The results obtained for 48 paired tissues revealed that APOBEC2 expression in stomach adenocarcinoma tissues was lower compared to that in the non-malignant gastric tissues (P < 0.0001; Fig. 1d). The expression of APOBEC2 was found to be significantly down regulated in stomach adenocarcinoma tissue compared to the non-malignant fundic gland mucosa tissues (*P* < 0.0001; Fig. 1e and f). The immunoreaction of the APOBEC2-positive cells in the body of the stomach and antrum was comparatively stronger and lighter (Fig. 2). Representative immunohistochemistry images of APOBEC2 staining in gastric cancer tissues as Fig. 2.

### Results of western bolt and qRT-PCR

**Fig. 3:**
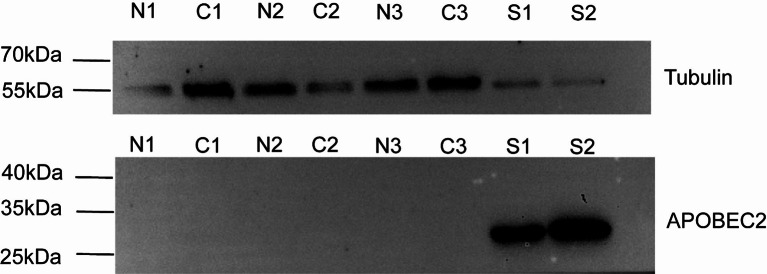
Western bolt analysis of APOBEC2 protein expression in skeletal muscle, STAD and adjacent normal gastric tissues. The upper panel was the result of western blot using anti-tubulin, while the lower panel using anti-APOBEC2. Samples in upper panel and lower panel both include normal gastric tissues (N1, N2 and N3), stomach adenocarcinoma tissues (C1, C2 and C3), and skeletal muscle tissues (S1 and S2). Of note, skeletal muscle tissues served as positive control

The results of western bolt showed that APOBEC2 protein of molecular weight about 26 kDa was detected in human skeletal muscle. However, little or no APOBEC2 expression was detected in STAD and adjacent normal tissues by western blot. Results of western bolt using anti-APOBEC2 and anti-tubulin as Fig. 3. The results of qRT-PCR demonstrated that APOBEC2 mRNA could be detected in gastric cancer cell line HGC27 and AG5 (see Fig. S2). In addition, the results obtained for 8 paired tissues revealed that relative APOBEC2 mRNA in stomach adenocarcinoma tissues was lower compared to that in the non-malignant gastric tissues (see Fig. S2).

### Relationship of APOBEC2 with CD66b, CD163 and other clinicopathological variables

APOBEC2 expression was detected in 339 of the cases accounting for 68.3% of all cases (Table 1). Among all specimens, the median count of CD66b^+^ TANs was 21, whereas the median count of CD163^+^ tumor-associated macrophages was 126. APOBEC2 was significantly related with CD66b (*P* = 0.001), differentiation grade (*P* < 0.001), TNM stage (*P* = 0.003), WHO histological classification (*P* < 0.001), Lauren histological type (*P* < 0.001) and gender (*P* = 0.004). There was no statistical correlation between APOBEC2 and CD163. We also explored the relationships of APOBEC2 with tumor size, location, serum CA19-9 levels and serum CEA levels in 496 cases, although statistical correlation was not evidenced (Table 1).

**Table 1:** Association of APOBEC2 expression with the demographic and clinical variables of STAD

Variables	Total(n)	APOBEC2 Negative(n, %)	APOBEC2 Positive(n, %)	*P* value*
Age(median,58years)				
≤ 58	250	81 (32.4)	169 (67.6)	0.718
> 58	246	76 (30.9)	170 (69.1)	
**Gender**				
Male	355	99 (27.9)	256 (72.1)	0.004
Female	141	58 (41.1)	83 (58.9)	
**Size(median,4.0 cm)**				
≤ 4.0	310	99 (31.9)	211 (68.1)	0.861
> 4.0	186	58 (31.2)	128 (68.8)	
**Differential grade**				
Well	7	1 (14.3)	6 (85.7)	< 0.001**
Moderate	228	26 (11.4)	202 (88.6)	
Poor	261	130 (49.8)	131 (50.2)	
**WHO histologic type**				
Tubular	319	47 (14.7)	272 (85.3)	< 0.001
Papillary	9	1 (11.1)	8 (88.9)	
Mucinous	18	14 (77.8)	4 (22.2)	
Poorly cohesive	150	95 (63.3)	55 (36.7)	
**Lauren’s type**				
Intestinal	328	48 (14.6)	280 (85.4)	< 0.001
Diffuse	168	109 (64.9)	59 (35.1)	
**TNM stage**				
I	160	35 (21.9)	125 (78.1)	0.003**
II	134	48 (35.8)	86 (64.2)	
III	195	70 (35.9)	125 (64.1)	
IV	7	4 (57.1)	3 (42.9)	
**Chemotherapy**				
Yes	339	116 (34.2)	223 (65.8)	0.071
No	157	41 (26.1)	116 (73.9)	
**Serum CEA (ng/ml)**				
< 5	384	122 (31.8)	262 (68.2)	0.833
≥ 5	81	24 (29.6)	57 (70.4)	
Missing	31	11 (35.5)	20 (64.5)	
**Serum CA199(U/ml)**				
< 37	380	120 (31.6)	260 (68.4)	0.984
≥ 37	64	20 (31.3)	44 (68.8)	
Missing	52	17 (32.7)	35 (67.3)	
**CD66b(median,21)**				
≤ 21	255	98 (38.4)	157 (61.6)	0.001
> 21	241	59 (24.5)	182 (75.5)	
**CD163(median,126)**				
≤ 126	249	80 (32.1)	169 (67.9)	0.819
> 126	247	77 (31.2)	170 (68.8)	

*Chi square test or Fisher’s exact test; **Mann-Whitney U test (non-parametric)

### Prognosis value of APOBEC2 in STAD

**Fig. 4:**
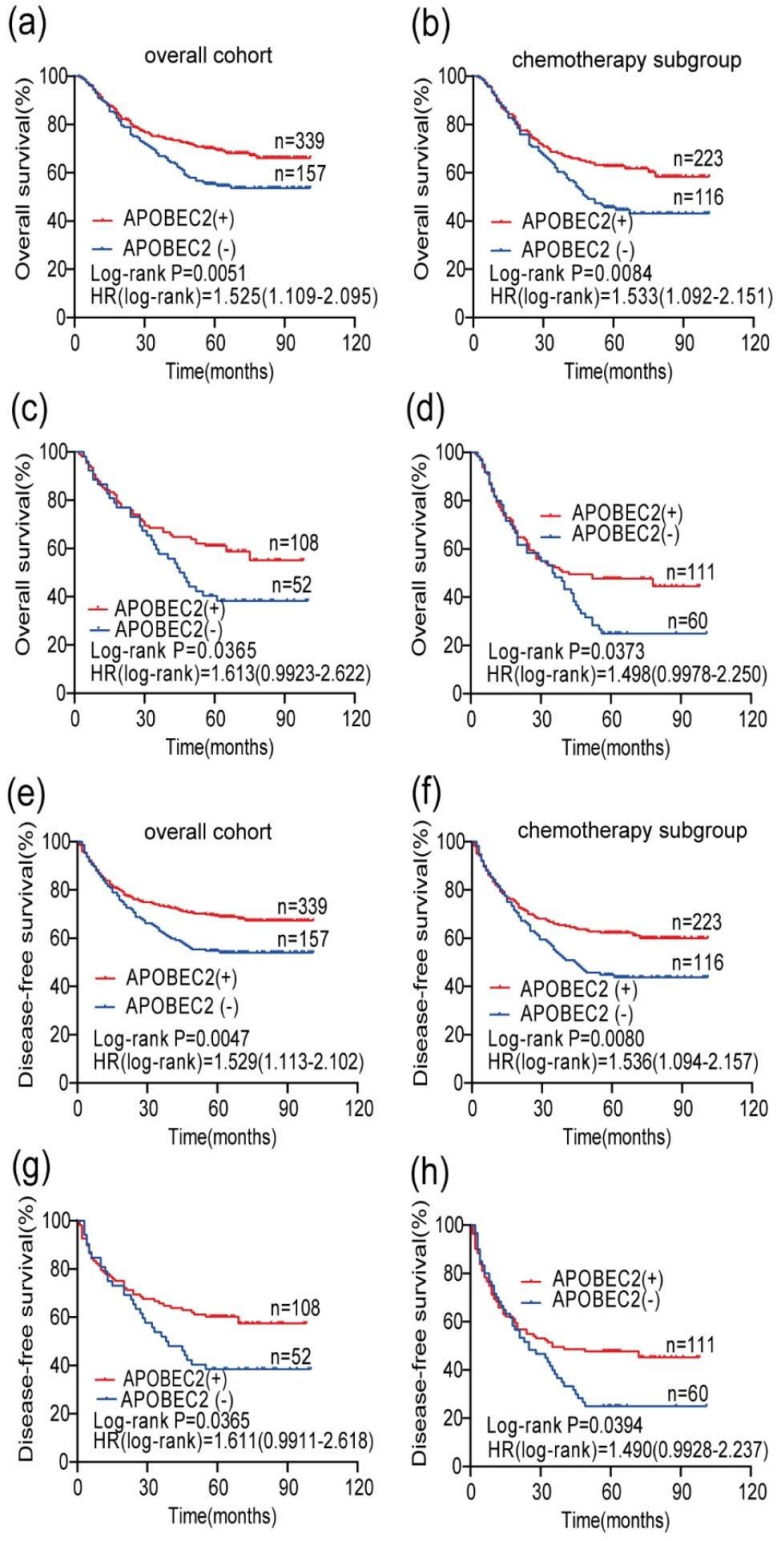
The survival curve comparing patients with APOBEC2-positive(red) and APOBEC2-negative(blue): (**a**) Overall survival (OS) curve of patients in overall cohort(n = 496); (**b**) OS curve of patients receiving postoperative chemotherapy (n = 339); (**c**) OS curve of patients larger than the median age and receiving postoperative chemotherapy (n = 160); (**d**) OS curve of stage III patients who received postoperative chemotherapy (n = 171); (**e**) Disease-free survival (DFS) curve of patients in overall cohort(n = 496); (**f**) DFS curve of patients receiving postoperative chemotherapy (n = 339); (**g**) DFS curve of patients larger than the median age and receiving postoperative chemotherapy (n = 160); (**h**) DFS curve of stage III patients who received postoperative chemotherapy (n = 171)

In overall cohort, Kaplan-Meier survival analyses of OS and DFS indicated that the APOBEC2-negative group had worse OS and DFS compared with APOBEC2-positive group (*P* = 0.0051 and *P* = 0.0047, respectively, Fig. 4a and e).

In our cohort, the number of patients receiving postoperative chemotherapy was 339. To confirm the prognostic impact of the APOBEC2 in patients received postoperative chemotherapy, we performed the survival analyses. Kaplan-Meier survival analyses revealed that both OS (Hazard Ratio 1.533, 95%CI1.092–2.151, *P* = 0.0084) and DFS (Hazard Ratio 1.536, 95%CI1.094–2.157, *P* = 0.0080) were significantly shorter in the APOBEC2-negative group than in the APOBEC2-positive group (Fig. 4b and f). In the subset of patients larger than the median age (58 years) who received postoperative chemotherapy (n = 160), we still found a significant difference in OS (Hazard Ratio 1.613, 95%CI0.9923–2.622, *P* = 0.0365) and DFS (Hazard Ratio 1.611, 95%CI0.9911–2.618, *P* = 0.0365) between the APOBEC2-positive group and the APOBEC2-negative group (Fig. 4c and g). However, in the subset of patients below median age (58 years) who received postoperative chemotherapy (n = 179), there was no significant difference in OS (Hazard Ratio 1.486, 95%CI0.9231–2.391, *P* = 0.0846) and DFS (Hazard Ratio 1.529, 95%CI0.9469–2.469, *P* = 0.0824) between the APOBEC2 positive group and negative group (Fig. S3). Furthermore, among stage III patients who received postoperative chemotherapy (n = 171), we found a significant difference in OS (Hazard Ratio 1.498, 95% CI 0.9978–2.250, *P* = 0.0373) and DFS (Hazard Ratio 1.490, 95% CI 0.9928–2.237, *P* = 0.0394) between the APOBEC2-positive group and the APOBEC2-negative group (Fig. 4d and h). However, among stage II patients who received postoperative chemotherapy (n = 109), no significant difference in OS or DFS was found between the APOBEC2-positive group and the APOBEC2-negative group (Fig. S3).

However, no significant difference in OS or DFS was found between the APOBEC2-positive group and the APOBEC2-negative group within the stage I + II, stage III + IV, moderately and highly differentiating, or poorly differentiating subsets (Fig. S3).

There were 8 clinicopathological factors shown in Table 2 that were investigated to determine whether they identified in the univariate analysis and were assessed in the multivariate analysis. In univariate Cox analysis, it is shown that OS and DFS in patients with STAD were associated with APOBEC2 expression status, size of tumor, neoplasm staging, and postoperative chemotherapy variables. However, in the multivariate Cox analysis, only size of tumor, neoplasm staging, and postoperative chemotherapy variables were still associated with OS and DFS, while not supporting APOBEC2 as an independent factor affecting OS and DFS in patients with STAD.

**Table 2:** Univariate and multivariate cox regression analysis of factors associated with OS and DFS

	Overall survival (OS)				Disease-free survival (DFS)			
	Univariate analysis of OS Multivariate analysis of OS				Univariate analysis of DFS Multivariate analysis of DFS			
**Variables**	HR (95% CI)	*P* value	HR (95% CI)	*P* value	HR (95% CI)	*P* value	HR (95% CI)	*P* value
**APOBEC2**								
Positive	0.656 (0.486,0.884)	0.006	0.816 (0.574,1.161)	0.259	0.653 (0.485,0.881)	0.005	0.821 (0.578,1.166)	0.270
Negative								
**Age (median,58 years)**								
> 58	1.277 (0.952,1.715)	0.103			1.278 (0.952,1.715)	0.103		
≤ 58								
**Gender**								
Female	1.233 (0.899,1.692)	0.194			1.221 (0.890,1.676)	0.215		
Male								
**Size(median,4.0 cm)**								
> 4.0	3.217 (2.386,4.335)	< 0.001	1.878 (1.361,2.593)	< 0.001	3.232 (2.398,4.357)	< 0.001	1.896 (1.375,2.615)	< 0.001
≤ 4.0								
**Differentiation grade**								
Poor	1.754 (1.294,2.377)	< 0.001	1.397 (0.948,2.059)	0.091	1.747 (1.290,2.368)	< 0.001	1.411 (0.958,2.080)	0.082
Well + moderate								
**Lauren’s histologic type**								
Diffuse	1.327 (0.983,1.792)	0.065	0.936 (0.615,1.426)	0.758	1.316 (0.975,1.777)	0.073	0.915 (0.602,1.389)	0.677
Intestinal								
**TNM stage**								
III + VI	4.867 (3.537,6.699)	< 0.001	3.215 (2.253,4.589)	< 0.001	4.870 (3.539,6.702)	< 0.001	3.205 (2.248,4.570)	< 0.001
I + II								
**Chemotherapy**								
No	0.336 (0.225,0.503)	< 0.001	0.648 (0.422,0.997)	0.049	0.335 (0.224,0.502)	< 0.001	0.643 (0.418,0.987)	0.044
Yes								

*HR* hazard ratio, *CI* confidence interval, *APOBEC2* apolipoprotein B mRNA editing enzyme catalytic polypeptide-like 2, *TNM* tumor node metastasis

### Combined prognostic value of APOBEC2 and CD66b in STAD

In the present study, we also attempt to evaluate the combined prognostic value of APOBEC2 and CD66b in STAD. We combined the APOBEC expression and the infiltration of CD66 + TANs as a two-marker predictor, which classified 496 patients into four subgroups: APOBEC2^−^CD66b^low^ (APOBEC2 negative and CD66b low), APOBEC2^−^CD66b^high^ (APOBEC2 negative and CD66b high), APOBEC2^+^CD66b^low^ (APOBEC2 positive and CD66b low), APOBEC2^+^CD66b^high^ (APOBEC2 positive and CD66b high).

In overall cohort, the two-marker predictor could stratify patients into different groups with distinct prognosis. Patients with APOBEC2^−^CD66b^low^ had the worst OS and DFS among all four subgroups, whereas patients with APOBEC2^+^CD66b^high^ had the best OS and DFS (Fig. S4). Univariate Cox analysis showed that patients subgroups with APOBEC2^−^CD66b^low^ (Hazard Ratio 1.867, 95%CI1.255–2.776, *P* = 0.002), patients subgroups with APOBEC2^−^CD66b^high^ (Hazard Ratio 1.596, 95%CI0.996–2.556, *P* = 0.052), patients subgroups with APOBEC2^+^CD66b^low^ (Hazard Ratio 1.348, 95%CI0.923–1.970, *P* = 0.122) were gradually associated with shorter OS when compare with the patients subgroups with APOBEC2^+^CD66b^high^ (Table 3). Univariate Cox analysis showed that patients subgroups with APOBEC2^−^CD66b^low^ (Hazard Ratio 1.869, 95%CI1.256–2.779, *P* = 0.002), patients subgroups with APOBEC2^−^CD66b^high^ (Hazard Ratio 1.622, 95%CI1.013–2.598, *P* = 0.044), patients subgroups with APOBEC2^+^CD66b^low^ (Hazard Ratio 1.360, 95%CI0.931–1.987, *P* = 0.112) were gradually associated with shorter DFS when compare with the patients subgroups with APOBEC2^+^CD66b^high^ (Table 3). However, in the multivariate Cox analysis, only TNM stage, size, and postoperative chemotherapy variables were still associated with OS and DFS, while not supporting the two-marker predictor as an independent factor affecting OS and DFS in patients with STAD (Table 3). In addition, there was no statistically significant difference in OS or DFS between the four groups within the stage I/II, stage III/IV, well/moderately differentiating or poorly differentiating categories (Fig. S4).

**Table 3:** Univariate and multivariate cox regression analysis of factors associated with OS and DFS

	Overall survival (OS)				Disease-free survival (DFS)			
	Univariate analysis of OS Multivariate analysis of OS				Univariate analysis of DFS Multivariate analysis of DFS			
**Variables**	HR (95% CI)	*P* value	HR (95% CI)	*P* value	HR (95% CI)	*P* value	HR (95% CI)	*P* value
**APOBEC2(A) and CD66b(B)**								
A^+^ and B high	Ref							
A- and B low	1.867 (1.255,2.776)	0.002	1.267 (0.807,1.990)	0.304	1.869 (1.256,2.779)	0.002	1.248 (0.795,1.961)	0.336
A- and B high	1.596 (0.996,2.556)	0.052	1.436 (0.865,2.383)	0.162	1.622 (1.013,2.598)	0.044	1.497 (0.904,2.479)	0.117
A^+^ and B low	1.348 (0.923,1.970)	0.122	0.924 (0.607,1.409)	0.715	1.360 (0.931,1.987)	0.112	1.183 (0.807,1.733)	0.389
**Age (median,58 years)**								
> 58	1.277 (0.952,1.715)	0.103			1.278 (0.952,1.715)	0.103		
≤ 58								
**Gender**								
Female	1.233 (0.899,1.692)	0.194			1.221 (0.890,1.676)	0.215		
Male								
**Size(median,4.0 cm)**								
> 4.0	3.217 (2.386,4.335)	< 0.001	1.877 (1.358,2.594)	< 0.001	3.232 (2.398,4.357)	< 0.001	1.898 (1.374,2.622)	< 0.001
≤ 4.0								
**Differentiation grade**								
Poor	1.754 (1.294,2.377)	< 0.001	1.407 (0.954,2.075)	0.085	1.747 (1.290,2.368)	< 0.001	1.424 (0.966,2.099)	0.074
Well + moderate								
**Lauren’s histologic type**								
Diffuse	1.327 (0.983,1.792)	0.065	0.924 (0.607,1.409)	0.715	1.316 (0.975,1.777)	0.073	0.904 (0.595,1.373)	0.634
Intestinal								
**TNM stage**								
III + VI	4.867 (3.537,6.699)	< 0.001	3.206 (2.244,4.581)	< 0.001	4.870 (3.539,6.702)	< 0.001	3.207 (2.246,4.578)	< 0.001
I + II								
**Chemotherapy**								
No	0.336 (0.225,0.503)	< 0.001	0.648 (0.421,0.996)	0.048	0.335 (0.224,0.502)	< 0.001	0.642 (0.418,0.986)	0.043
Yes								

HR, hazard ratio; CI, confidence interval; APOBEC2, apolipoprotein B mRNA editing enzyme catalytic polypeptide-like 2; A+,APOBEC2 positive; A-,APOBEC2,negative; B high,CD66b high; B low,CD66b low; Ref, reference; TNM, tumor node metastasis

## Discussion

In this study, we noticed the frequency of APOBEC2 gene alteration in stomach adenocarcinoma using the database from cBioportal wanderer platform. It showed that the proportion of APOBEC2 gene alteration was about 5%, among these only 1 of 89 Asian patients existed APOBEC2 gene alteration. Similarly, a few previous studies have reported the APOBEC2 gene is conserved [20]. These findings seemed to be consistent with our observation. Furthermore, we performed the analysis with cancer tissues in GEPIA database and revealed APOBEC2 gene expression is commonly down regulated in cancer tissues compared with normal tissues, suggesting APOBEC2 may be associated with the development of stomach adenocarcinoma.

Next, we investigated the expression of APOBEC2 protein in gastric cancer tissue and non-malignant tissues. We noticed that the obvious difference of distribution pattern of APOBEC2 between in gastric cancer tissue and non-malignant tissues. Notably, APOBEC2 was positively stained in the fundic gland, whereas stain of pyloric gland was negative (Fig. 2). Our proteomics and microarray data were consistent with the finding that the fundus and body of stomach had a certain level of APOBEC2 mRNA [21]. Gastric fundus glands, mostly composed of parietal cells that generate hydrochloric acid and chief cells that secrete pepsinogen, are distributed at the gastric body and fundus. These products of the gastric mucosa such as stomach acid and pepsinogen have been confirmed to have important functions in screening, diagnosis and prognosis of gastric disease. To prove that the expression of APOBEC2 in gastric fundus glands might play an important role in the physiological functions of stomach. As for the result of western bolt, we can’t find the significant APOBEC2 protein expression in fresh gastric tissues, even we can show the APOBEC2 expression samples in striated muscle tissues, it hints us that APOBEC2 protein expression levels are relatively low in clinic samples, especially considering the detected mixture samples including the uncertain exact quantity and ratios cancer tissues and the non-cancer tissues. To research the expression of APOBEC2 protein in STAD and normal gastric tissues better requires us to use laser microdissection.

We showed that the expression of APOBEC2 was associated with the infiltration of CD66b^+^TANs, differentiation grade, TNM stage, histological classification, and gender in STAD. In particular, the percentage of APOBEC2-positive patients in the well differentiation grade was high. Furthermore, the Kaplan-Meier analyses in 496 patients with STAD also indicated that APOBEC2-negative patients had higher risk of death and recurrence compared with APOBEC2-positive patients. The above results indicated that APOBEC2 may be involved in the progression and prognosis of gastric adenocarcinoma. Although APOBEC2 expression was associated with the infiltration of CD66b^+^ TANs in STAD, there was no correlation between the infiltration of CD163^+^ TAMs. Consequently, there are questions to be answered about the exact mechanism of correlation between APOBEC2 and tumor infiltrating immune cell. Some studies suggested that the APOBEC2 might be involved in immune reactions to infection or inflammation. For example, it has been reported that elevated APOBEC2 was negatively related with serum IgG level in the tonsils with IgA nephritic patients [22]. Moreover, Matsumoto et al. reported that pro-inflammatory cytokines (TNF-α and IL-1β) can promote the recruitment of NF-kB, which is known to have a complicate relationship with carcinogenesis among several functional NF-kB binding sties in the APOBEC2 promoter region [23]. Research showed that neutrophils can produce TNF-α and IL-1β involved in in the inflammatory response and immune regulation [24]. Further studies are warranted to clarify associations between APOBEC2 and tumor microenvironment including immune cell infiltration.

At present, adjuvant chemotherapy is usually recommended for patients with advanced gastric cancer, according to the guidelines of the National Comprehensive Cancer Network (NCCN) [25]. In addition, a certain number of trials around the world have shown that adjuvant chemotherapy contributes to reducing the rate of recurrence and improving survival rates in gastric cancer [26]. However, given the adverse reactions and resistance issue, it is critical to look for biomarkers that might predict adjuvant chemotherapy success. In our study, Kaplan-Meier survival curves for the patients receiving postoperative chemotherapy (n = 339) showed a difference between the APOBEC2-negative arm and the APOBEC2-positive arm. Here, we showed that APOBEC2-negative was related to poor survival outcomes in patients receiving postoperative adjuvant chemotherapy. Furthermore, we also illustrated the remarkable survival difference in stage III patients who received postoperative chemotherapy (n = 171). As for the results of stage II patients, we speculated that the small sample size was under-powered to show statistical differences between groups. To reduce the impact of age, we performed survival analyses on patients larger than the median age (n = 160) and on patients below the median age (n = 179) who received postoperative chemotherapy, respectively. Then the remarkable survival difference could still be found. So, we presume that detection of APOBEC2 expression could predict the efficacy of postoperative chemotherapy in patients. TMB was negatively correlated with APOBEC2 expression in STAD [12]. Patients with TMB-low benefited better from postoperative chemotherapy in terms of OS and DFS in some cohorts [27]. Combine our findings, it may serve as an inspiration for future research on the influence of postoperative chemotherapy, APOBEC2, and TMB on the prognosis of STAD patients.

Additionally, we also evaluated the influence of postoperative chemotherapy on prognosis of gastric cancer. Multivariate analysis revealed that received postoperative chemotherapy was associated with poor prognosis in that patients with adjuvant chemotherapy had higher risk of death and recurrence compared patients without adjuvant chemotherapy (Tables 2 and 3). These unexpected results were similar to those earlier published studies that revealed a greater survival rate in the surgery-only control group and no statistically significant benefit from adjuvant chemotherapy. For example, the median overall survival was 57.6 months and 56.7 months, respectively, in a study by the Italian Oncology Group for Cancer Research that compared surgery alone vs. adjuvant cisplatin chemotherapy [28]. In another randomized trial designed by the European Organization for Research and Treatment of Cancer (EORTC) institutions, there were no significant differences between the postoperative chemotherapy arm and control arm (treated with surgical operation alone) for either OS or DFS [29]. They reported 5-year DFS was 41% in the treatment arm and 42% in the control arm, and 5-year OS was 43% and 44%, respectively. Furthermore, we speculated that the unexpected results of our cohort may be because of the different chemotherapy regimen, poor compliance to adjuvant chemotherapy or elderly patients combined with poor chemotherapy tolerance.

In our study, univariate Cox analysis (Table 2) revealed that negative APOBEC2 was associated with poor prognosis in that APOBEC2-negative patients had higher risk of death and recurrence compared with APOBEC2-positive patients. When used together as a two-marker predictor, APOBEC2 and CD66b were able to separate 496 patients with STAD into groups with different prognoses. Univariate Cox analysis revealed that patients with APOBEC2^−^CD66b^low^ had the worst OS and DFS among all four subgroups (Table 3). Nevertheless, it is noted that when adjusted for other factors (e.g., TNM stage) in the multivariate Cox analysis, APOBEC2 was not an independent prognostic factor for STAD. And multivariate Cox analysis demonstrated that the two-marker classifier (APOBEC2 and CD66b) couldn’t independently predict the prognosis of STAD, when the APOBEC2^+^CD66b^high^ subgroup was used to a reference. In a word, we failed to show APOBEC2 or the combination of APOBEC2 and CD66 was an independent prognostic factor for stomach adenocarcinoma. There are several explanations for the unexpected results. First, we speculated that the relatively low proportion of early disease in our cohort might affect the prognostic evaluation values of APOBEC2. Second, there are other important factors that influence the recurrence and the survival of STAD. Thirdly, it might be due to a lack of statistical power resulted from limited sample size (n = 496, particularly, there were some samples with censored data).

It should be noted that our study has certain limitations. More verification and molecular mechanisms are needed even we have made the connections that patients with positive APOBEC2 may benefit from postoperative chemotherapy to improve OS and DFS, and the APOBEC2 expression profiles were evaluated by TMAs, immunohistochemical, conventional pathological methods. High-throughput sequencing, such as single-cell RNA sequencing (scRNA-seq), has become a powerful tool in the field of cancer research and has provided novel insights into the cellular and molecular features of tumors [30–32]. Further studies are needed to clarify the expression of APOBEC2 in specific cells of gastric cancer based on scRNA-seq.

## Conclusion

In conclusion, our study has comprehensively explored the expression and clinical significance of APOBEC2 in STAD tissues and non-malignant tissues. We found that APOBEC2 was positively stained in the fundic gland, whereas staining of the pyloric gland was mostly negative. Although we failed to show that APOBEC2 was an independent predictor of overall survival and disease-free survival, we still found that the association among APOBEC2 and CD66b, differentiation grade, TNM stage, histological classification and gender. Particularly, our results suggested that patients with positive APOBEC2 can benefit from postoperative adjuvant chemotherapy. The combination of APOBEC2 and CD66b could further stratify patients into different groups with distinct prognoses.

### Electronic supplementary material

Below is the link to the electronic supplementary material.

Supplementary Material 1

Supplementary Material 2

## Data Availability

The publicly accessible databases supporting the conclusions of this article are available in hyperlink: https://www.cbioportal.org/results/cancerTypesSummary?cancer_study_list=stad_tcga2Cstad_utokyo2Cstad_pfizer_uhongkong2Cstad_oncosg_20182Cstad_uhongkongZ_SCORE_THRESHOLD=2.0RPPA_SCORE_THRESHOLD=2.0profileFilter=mutations2Cgisticcase_set_id=allgene_list=APOBEC2geneset_list=20tab_index=tab_visualizeAction=Submit; https://www.cbioportal.org/results/cancerTypesSummary?plots_horz_selection=7B7Dplots_vert_selection=7B7Dplots_coloring_selection=7B7Dtab_index=tab_visualizeAction=Submitsession_id=64d5f2021095f74ff797ee4a; http://gepia.cancer-pku.cn/detail.php?gene=APOBEC2. Other data used and/or analyzed during this study are available from the corresponding author upon reasonable request.

## Abbreviations

APOBEC2: Apolipoprotein B mRNA editing enzyme catalytic polypeptide-like 2
CNAs: Copy number alterations
CA199: Carbohydrate antigen 199
CEA: Carcinoembryonic antigen
CI: Confidence interval
DFS: Disease-free survival
EORTC: European organization for research and treatment of cancer
FFPE: Formalin-fixed paraffin-embedded
GC: Gastric cancer
GEO: Gene expression omnibus
GTEx: Genotype tissue expression
HR: Hazard ratio
H&E: Hematoxylin-eosin
iCGC: International cancer genome consortium
NCCN: National comprehensive cancer network
OS: Overall survival
TCGA: The cancer genome atlas
TMB: Tumor mutational burden
TMAs: Tissue microarrays
TNM: Tumor node metastasis
TANs: Tumor-associated neutrophils
TAMs: Tumor-associated macrophages
STAD: Stomach adenocarcinoma
