# Supplementary material for: Expression and prognostic value of APOBEC2 in gastric adenocarcinoma and its association with tumor-infiltrating immune cells
Source: BMC Cancer. 2024 Jan 2;24:15. doi: 10.1186/s12885-023-11769-3 (PMC10763203; doi:10.1186/s12885-023-11769-3)
Supplement: Supplementary file 1 — Supplementary Material 1 [file 12885_2023_11769_MOESM1_ESM.docx]

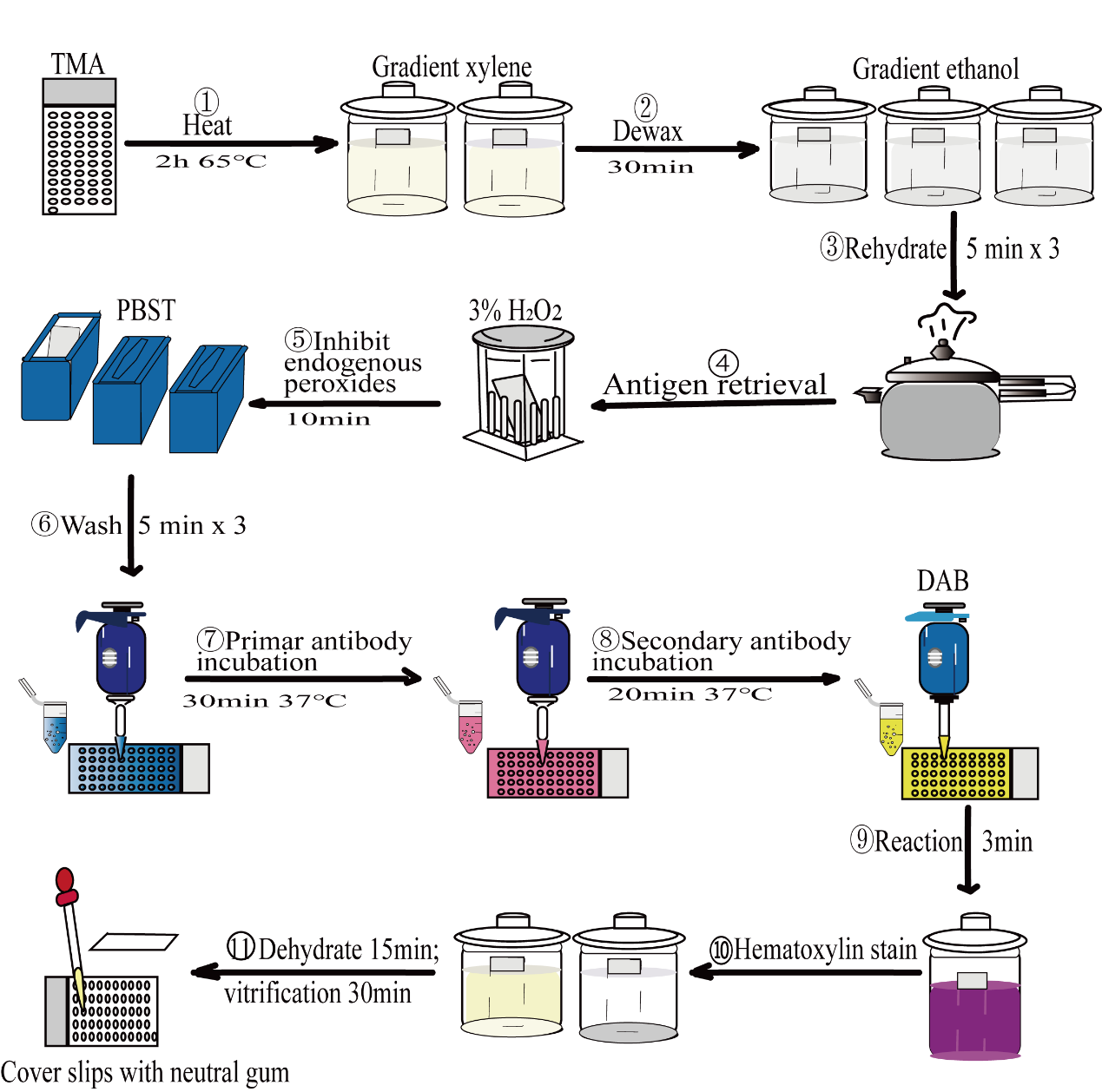


**Figure S1. The brief experimental process of immunohistochemical staining:** ①Array slide was heated for 2 hours at 65℃ ,and then ②dewaxed with grader xylene and ③rehydrated with grader ethanol. ④Antigen retrieval was performed by immersing the slides in 65℃ EDTA and boiling in a pressure cooker until blowing about 2min. ⑤Array slide was immersed in 3% H2O2 for 10 min to inhibit endogenous peroxides. ⑥Wash three times with PBST (Phosphate Buffer Solution with Tween) to 5 min each. ⑦Primary antibody incubation for 30min at 37℃. ⑧Secondary antibody incubation for 20 min at 37℃. ⑨Add 3-3'-diamino-benzidine (DAB) solution for 3min and then washing with tap water. ⑩Hematoxylin staining for 30sec and adding a step of hydrochloric acid alcohol differentiation for 2-3sec and blue liquid for 2min. ⑪Dehydrate using graded ethanol and vitrification by dimethylbenzene. Caution: Before or after dropping antibodies, the array slide must be washed three times with PBST.


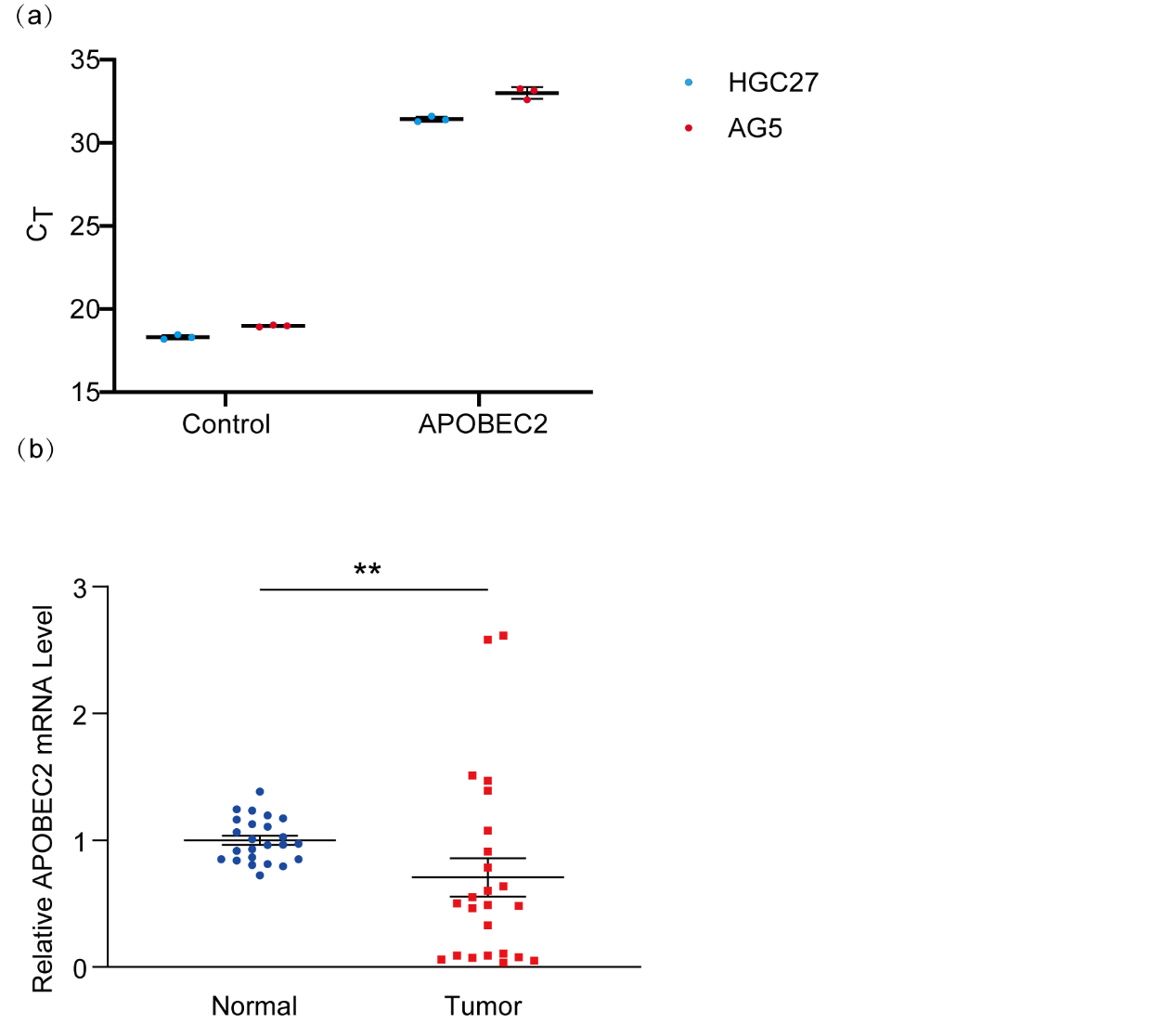


**Figure S2.** **Verification of differentially expressed APOBEC2 in gastric cancer cell line, STAD and normal tissues by qRT-PCR.** (a) Results of qRT-PCR of gastric cancer cell line HCG27 and AG5. All samples were measured in triplicate. (b) Relative APOBEC2 mRNA level in matched normal and malignant tissues (n=8). All samples were measured in triplicate.


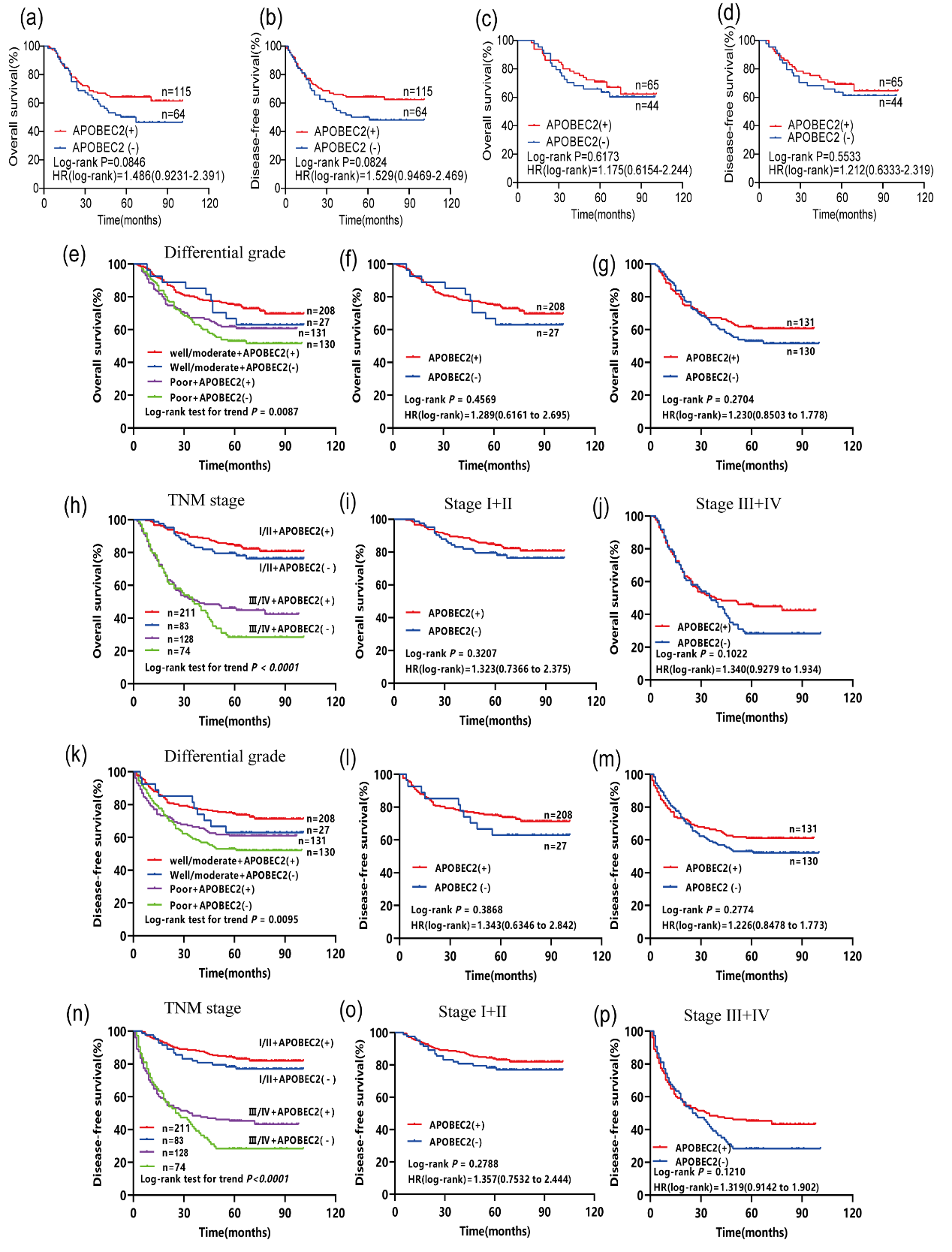


**Figure S3. The survival curve comparing patients with APOBEC2-positive and APOBEC2-negative among different subgroups.** (a,c) Overall survival (OS) and disease-free survival (DFS) curve of patients in the subset of patients below median age (58 years) who received postoperative chemotherapy (n=179). (b,d) OS and DFS curve of patients in stage II patients who received postoperative chemotherapy (n=109). (e,k) OS) and DFS curve of patients in differential grade (n=496). (f,i) OS and DFS curve in patients with well or moderate differentiation status (n=235). (g,m) OS and DFS curve in patients with poor differentiation status (n=261). (h,n) OS and DFS curve of patients in differential TNM stage (n=496). (i,o) OS and DFS curve in stage Ⅰ and stage II patients (n=294). (j,p) OS and DFS curve in stage Ⅲ and stage Ⅳ patients (n=202).


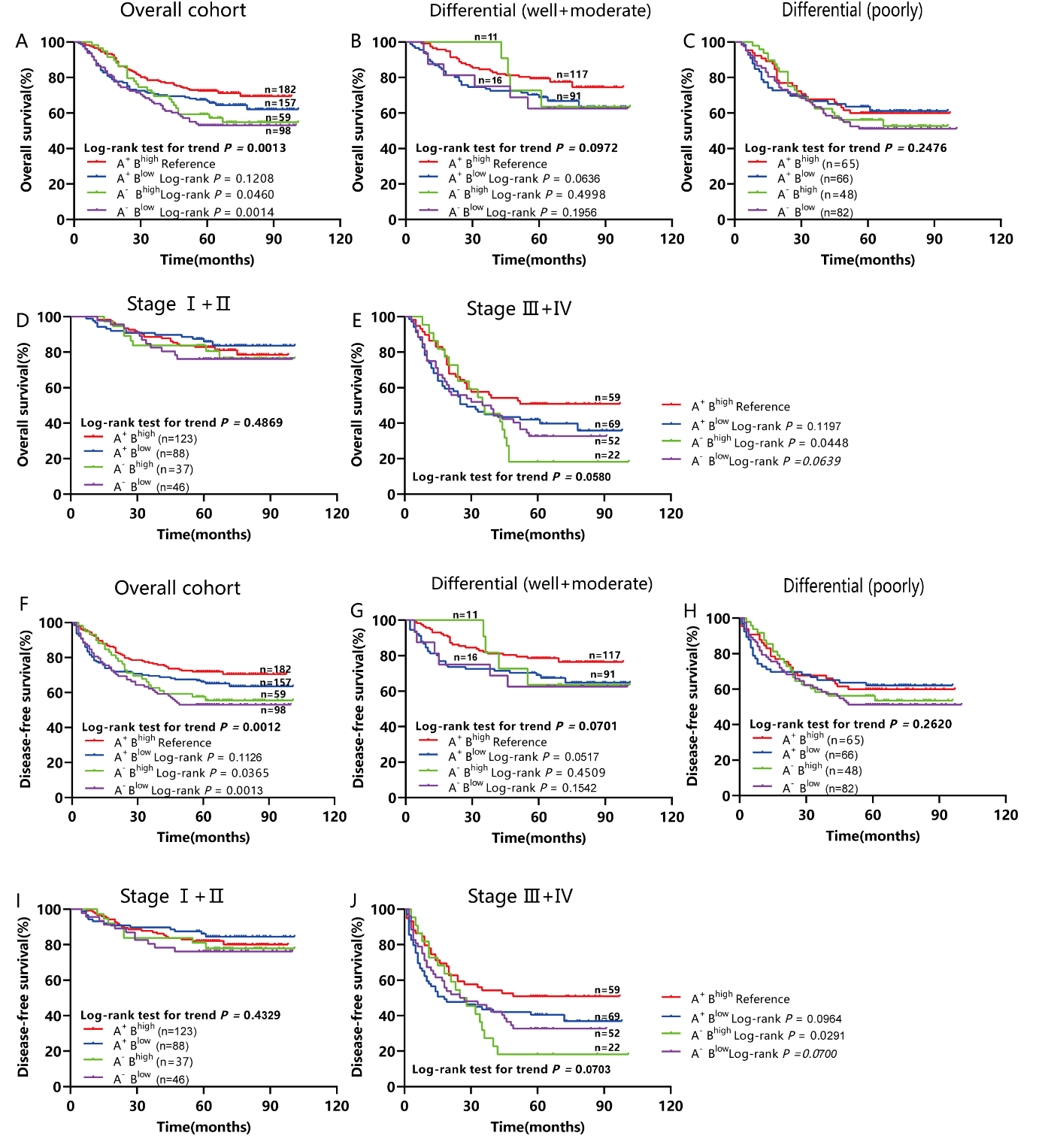


**Figure S4. The survival curve comparing patients with APOBEC2^-^CD66b^low^ (purple), APOBEC2^-^CD66b^high^ (green), APOBEC2^+^CD66b^low^ (blue) and APOBEC2^+^CD66b^high^ (red).** (A,F) Overall survival (OS) and disease-free survival (DFS) curve of patients in overall cohort(n=496); (B,G) OS and DFS curve in patients with well or moderate differentiation status (n=235); (C,H) OS and DFS curve in patients with poor differentiation status (n=261); (D,I) OS and DFS curve in stage Ⅰ and stage II patients (n=294); (E,J) OS and DFS curve in stage Ⅲ and stage Ⅳ patients (n=202).
