# Supplementary figures and images for: Expression and prognostic value of APOBEC2 in gastric adenocarcinoma and its association with tumor-infiltrating immune cells
Source: BMC Cancer. 2024 Jan 2;24:15. doi: 10.1186/s12885-023-11769-3 (PMC10763203; doi:10.1186/s12885-023-11769-3)

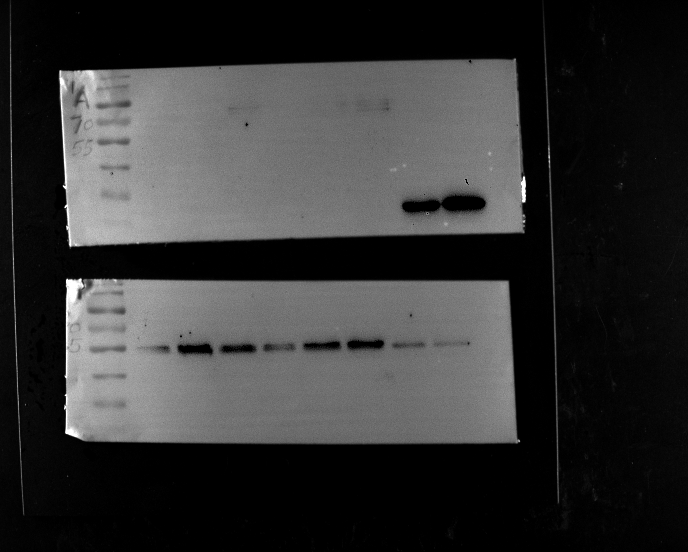

Supplement: Supplementary file 2 — Supplementary Material 2 [file 12885_2023_11769_MOESM2_ESM.tif]
